# Supplementary material for: Diversity and community structure of marine microbes around the Benham Rise underwater plateau, northeastern Philippines
Source: PeerJ. 2018 May 16;6:e4781. doi: 10.7717/peerj.4781 (PMC5960264; doi:10.7717/peerj.4781)
Supplement: Supplemental Information 1 — The depth profiles of physical (Figure S1) and chemical data (Figure S2) as well as rarefaction curves (Figure S3) are shown. Tables for the physical and chemical data used for Canonical Correspondence Analysis (Table S1), results of statistical tests (Table S4) and depth-associated OTUs (Table S5) are also shown. [file peerj-06-4781-s001.docx]

**Supplementary Materials**

**
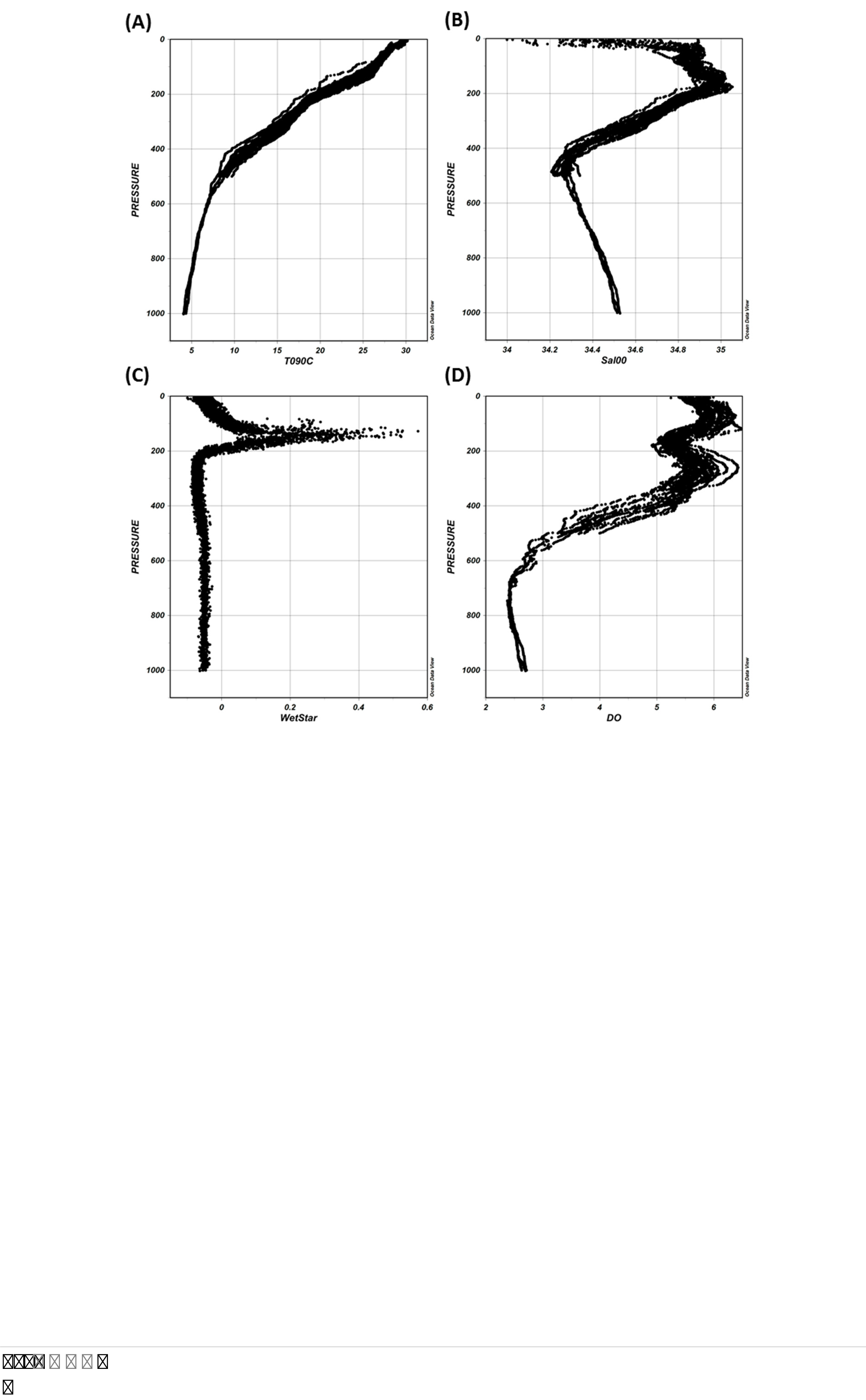
**

**Figure S1. Depth profiles for water parameters measured at sampling stations in Benham Rise.** (A) Temperature (T090C), (B) salinity (Sal00), (C) chlorophyll-a (WetStar), and (D) dissolved oxygen (DO).

**
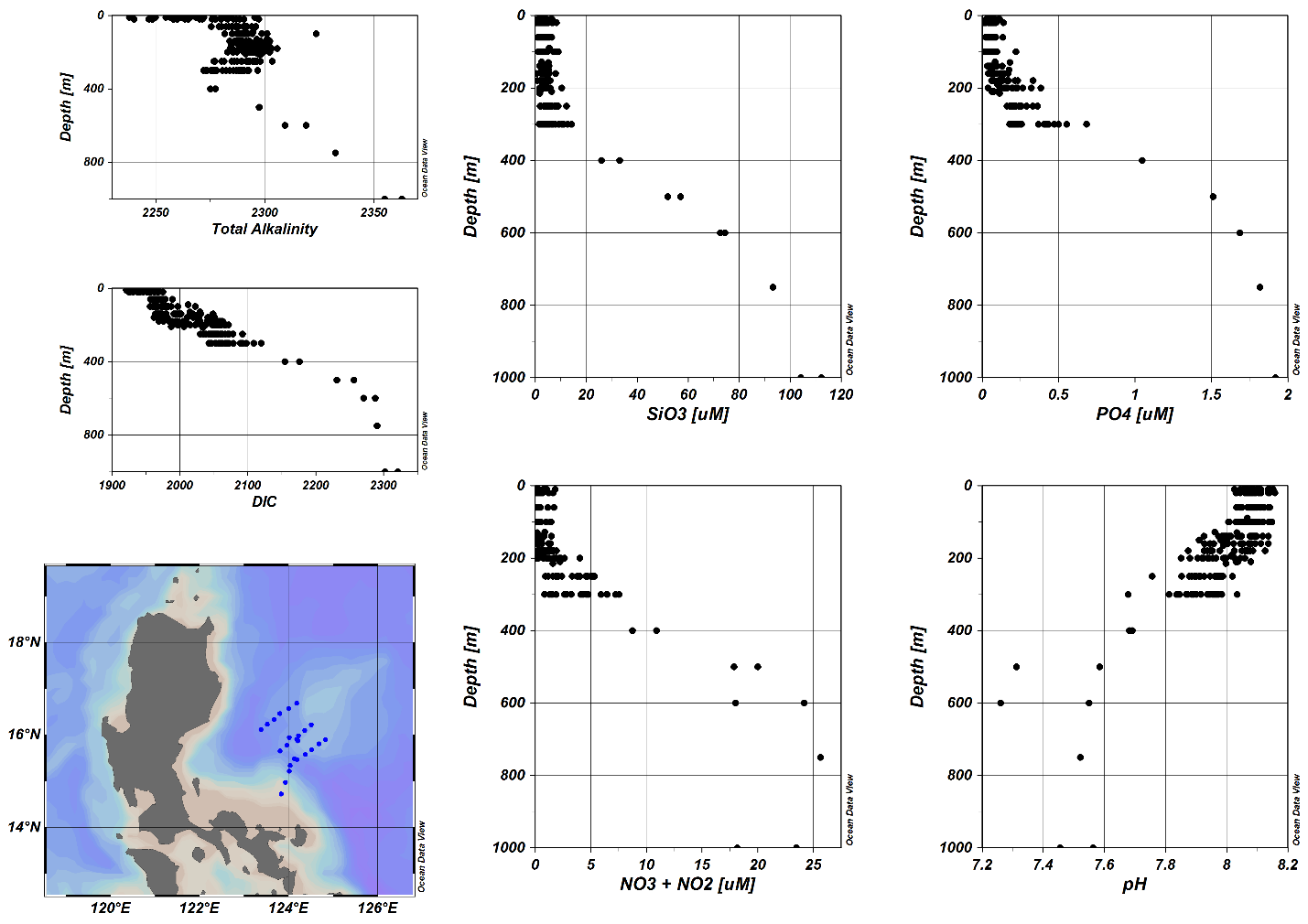
**

**Figure S2. Depth profiles of nutrients and carbonate parameters in Benham Rise**. Vertical profiles of chemical parameters for all 24 sampling stations (blue dots in map) covering ten depths per station.

**
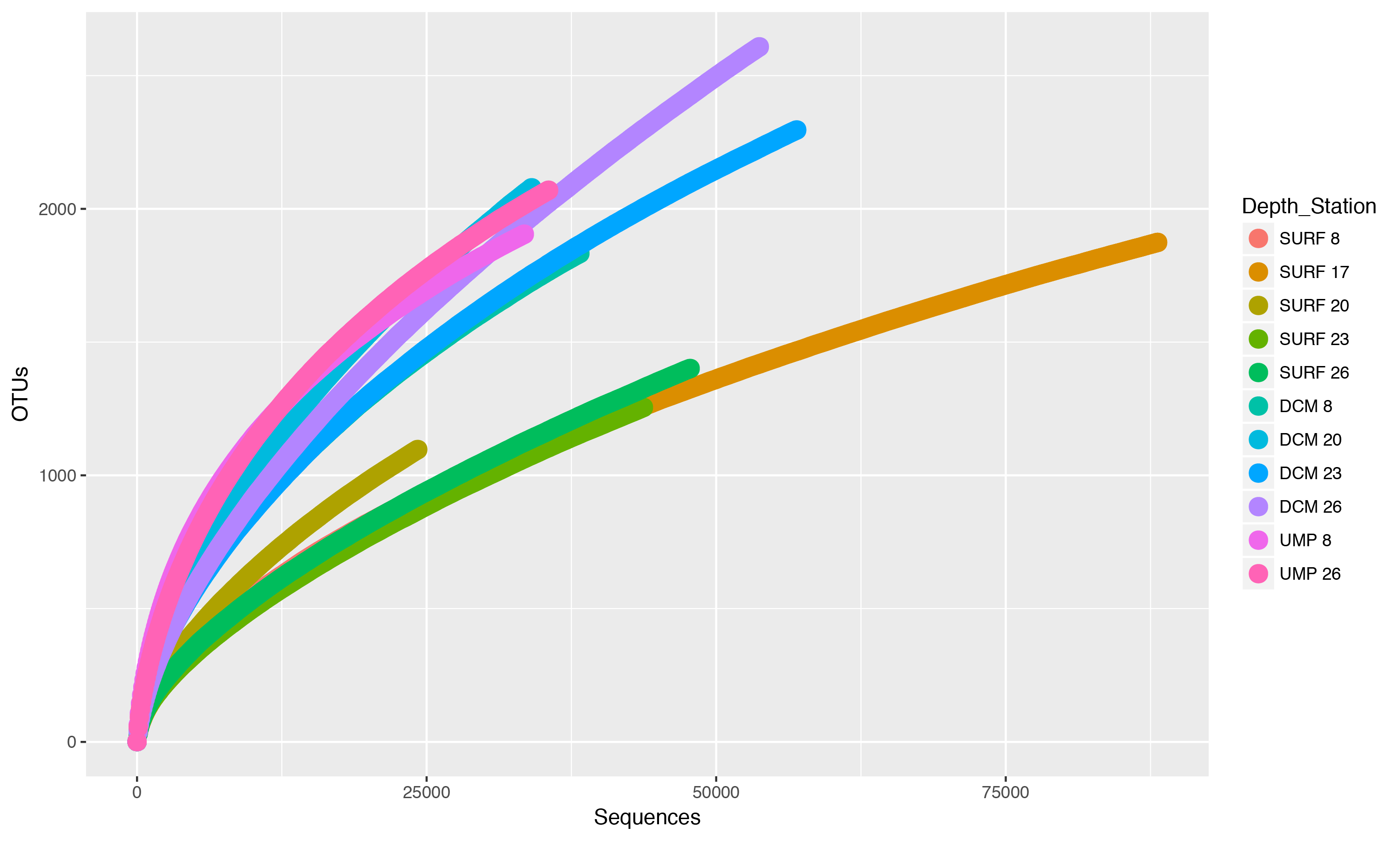
**

**Figure S3. Rarefaction curves for 16S rRNA libraries from the indicated sampling stations and depths.**

**Table S1.** Physical and chemical parameters of waters sampled for microbial analysis

| **Sample ID** | **Stn** | **Lat**  (deg) | **Long**  (deg) | **Depth** (m) | **Temperature**(^o^C) | **Salinity**  (psu) | **Turbidity**  (mg/L) | **Chl-a**  (mg/L) | **PO_4_** [uM] | **SiO_3_** [uM] | **NO_3_ + NO_2_** [uM] | **DO** [mg/L] | **Total Alkalinity** | **DIC** | **pH** |
| --- | --- | --- | --- | --- | --- | --- | --- | --- | --- | --- | --- | --- | --- | --- | --- |
| SURF 8 | 8 | 16.223 | 124.507 | 10 | 29.70 | 34.26 | 0.1217 | 0.00 | 0.11 | 1.16 | UD | 5.79 | 2250 | 1930 | 8.10 |
| SURF 17 | 17 | 16.232 | 123.519 | 10 | 29.75 | 34.62 | 0.1474 | 0.02 | 0.07 | 6.47 | UD | 5.54 | 2280 | 1963 | 8.06 |
| SURF 20 | 20 | 16.576 | 124.002 | 10 | 29.70 | 34.30 | 0.1157 | 0.00 | UD | 1.77 | UD | 5.64 | 2248 | 1938 | 8.09 |
| SURF 23 | 23 | 15.490 | 124.123 | 10 | 29.86 | 34.59 | 0.1161 | 0.00 | UD | 4.39 | UD | 5.61 | 2270 | 1951 | 8.07 |
| SURF 26 | 26 | 14.729 | 123.833 | 10 | 29.17 | 34.48 | 0.1306 | 0.00 | UD | 2.85 | UD | 5.81 | 2257 | 1933 | 8.14 |
| DCM 8 | 8 | 16.223 | 124.507 | 200 | 20.30 | 34.90 | 0.1234 | 0.00 | 0.15 | 1.84 | UD | 5.74 | 2292 | 1996 | 8.05 |
| DCM 20 | 20 | 16.576 | 124.002 | 160 | 24.58 | 34.92 | 0.1141 | 0.35 | 0.04 | 3.51 | 0.27 | 5.34 | 2290 | 2020 | 8.00 |
| DCM 23 | 23 | 15.490 | 124.123 | 140 | 24.63 | 34.97 | 0.1284 | 0.36 | UD | 2.74 | UD | 5.27 | 2302 | 2019 | 8.01 |
| DCM 26 | 26 | 14.729 | 123.833 | 90 | 24.51 | 34.94 | 0.1401 | 0.26 | UD | 5.57 | UD | 5.45 | 2295 | 2012 | 8.07 |
| UMP 8 | 8 | 16.223 | 124.507 | 300 | 16.60 | 34.67 | 0.1268 | 0.00 | 0.26 | 5.33 | UD | 5.34 | 2286 | 2053 | 7.94 |
| UMP 26 | 26 | 14.729 | 123.833 | 300 | 14.47 | 34.53 | 0.1378 | 0.00 | 0.42 | 14.26 | 7.23 | 5.03 | 2289 | 2109 | 7.86 |

legends: Stn – station, lat – latitude, long – longitude, deg – degree, UD – undetectable

**Table S2.** CTD data (see excel sheet)

**Table S3.** Chemical parameter data (see excel sheet)

**Table S4.** Statistical tests of microbial community structure similarity based on the Yue & Clayton and the Jaccard dissimilarity indices.

| **Parsimony test** | | | | | | | |
| --- | --- | --- | --- | --- | --- | --- | --- |
| Yue & Clayton | | | | Jaccard | | | |
| Tree# | Groups | ParsScore | ParsSig | Tree# | Groups | ParsScore | ParsSig |
| 1 | DCM-SURF | 4 | 1 | 1 | DCM-SURF | 1 | 0.009 |
| 1 | DCM-UMP | 1 | 0.128 | 1 | DCM-UMP | 1 | 0.142 |
| 1 | SURF-UMP | 1 | 0.113 | 1 | SURF-UMP | 1 | 0.128 |
| **Unweighted UniFrac** | | | | | | | |
| Yue & Clayton | | | | Jaccard | | | |
| Tree# | Groups | UWScore | UWSig | Tree# | Groups | UWScore | UWSig |
| 1 | DCM-SURF | 0.522015 | 0.793 | 1 | DCM-SURF | 1 | 0.036 |
| 1 | DCM-UMP | 1 | 0.332 | 1 | DCM-UMP | 1 | 0.37 |
| 1 | SURF-UMP | 1 | 0.282 | 1 | SURF-UMP | 1 | 0.047 |
| **Weighted UniFrac** | | | | | | | |
| Yue & Clayton | | | | Jaccard | | | |
| Tree# | Groups | WScore | WSig | Tree# | Groups | WScore | WSig |
| 1 | DCM-SURF | 0.326649 | 0.199 | 1 | DCM-SURF | 1 | <0.0010 |
| 1 | DCM-UMP | 1 | <0.0010 | 1 | DCM-UMP | 1 | 0.043 |
| 1 | SURF-UMP | 1 | <0.0010 | 1 | SURF-UMP | 1 | <0.0010 |
| **AMOVA** | | | | | | | |
| Yue & Clayton | | | | Jaccard | | | |
| DCM-SURF-UMP | Among | Within | Total | DCM-SURF-UMP | Among | Within | Total |
| SS | 1.34444 | 0.397095 | 1.74153 | SS | 1.32286 | 2.32542 | 3.64827 |
| df | 2 | 8 | 10 | df | 2 | 8 | 10 |
| MS | 0.672219 | 0.0496369 |  | MS | 0.661428 | 0.290677 |  |
| Fs: | 13.5427 |  |  | Fs: | 2.27547 |  |  |
| p-value: | 0.005 |  |  | p-value | <0.001 |  |  |
| **HOMOVA** | | | | | | | |
| Yue & Clayton | | | | Jaccard | | | |
|  | Bvalue | P-value | |  | BValue | P-value | |
| DCM-SURF-UMP | 3.11732 | 0.565 | | DCM-SURF-UMP | 0.0219614 | 0.191 | |
| **ʃ-LIBSHUFF** | | | | | | | |
| Yue & Clayton | | | | Jaccard | | | |
| Comparison | dCXYScore | Significance | | Comparison | dCXYScore | Significance | |
| DCM-SURF | 0.00651325 | 0.866 | | DCM-SURF | 0.1159202 | 0.0072 | |
| SURF-DCM | 0.00567132 | 0.7401 | | SURF-DCM | 0.08439142 | <0.0001 | |
| DCM-UMP | 0.83380875 | 0.0679 | | DCM-UMP | 0.07416752 | 0.0692 | |
| UMP-DCM | 0.25853403 | 0.5279 | | UMP-DCM | 0.13417697 | 0.2675 | |
| SURF-UMP | 0.80135013 | <0.0001 | | SURF-UMP | 0.13425305 | 0.0499 | |
| UMP-SURF | 0.23286927 | 0.6656 | | UMP-SURF | 0.22891821 | 0.0499 | |

**Table S5. Depth-associated OTUs.** Selected OTUs that differentiate between depths were identified using LEfSe. Selected OTUs responsible for differences in the grouping of samples were identified using indicator analysis.

| **OTU Number** | **Taxonomy** | **Depth** | **Test** | **p-value** |
| --- | --- | --- | --- | --- |
| Otu00005 | Nitrosopumilaceae | UMP | LEfSe | 0.012 |
| Otu00007 | SAR86 | SURF | LEfSe | 0.013 |
| Otu00009 | Marine Group II | DCM | LEfSe | 0.023 |
| Otu00015 | SAR116 | SURF | LEfSe | 0.014 |
| Otu00310 | Nitrospina | UMP | Indicator | 0.019 |
| Otu00702 | Spirochaeta | UMP | Indicator | 0.026 |
